# Running During Encoding Improves Word Learning for Children

**DOI:** 10.3389/fpsyg.2020.00684

**Published:** 2020-04-21

**Authors:** Gianluca Amico, Sabine Schaefer

**Affiliations:** Department of Sport Sciences, Saarland University, Saarbrücken, Germany

**Keywords:** physical exercise, acute physical exercise, lifespan, word learning, vocabulary, memory

## Abstract

The learning of new information is an important task in everyday life, especially at a young age. Acute physical exercise can facilitate cognitive processes in multiple ways, and previous studies have shown that memory can profit from physical exercise before and during the encoding of vocabulary. The current study investigates the interplay of movement and vocabulary learning and also addresses lifespan differences in these effects. Participants were recruited in a recreational basketball club. Children (*n* = 24, *M*_*age*_ = 12.3 years; 13 girls), young adults (*n* = 30, *M*_*age*_ = 21.5 years; 17 women), and older adults (*n* = 24, *M*_*age*_ = 59.3 years; 9 women) learned 20 new pseudo-words, which corresponded to a German word. In a between-subjects design, encoding took place either while standing, while running, or while running and dribbling a basketball. Recall was assessed three times throughout the learning session and on the following day. In children, more words could be remembered in the running condition compared to the standing condition. There were no differences between conditions for the young and older adults. Age-dependent reasons for this pattern of results are discussed and embedded into the literature of physical exercise. Our result suggests that implementing learning activities into children’s physical education or exercise activities could be beneficial.

## Introduction

The learning of new information is an important task in everyday life. Physical exercise can improve cognitive performances, which has been demonstrated for long-term as well as acute exercise interventions. Chronic physical exercise has been shown to enhance cognitive processes in multiple ways [see a review by Tomporowski et al. (2008) and a meta-analysis by Verburgh et al. (2014)]. In addition, chronic physical activity can support physical and cognitive development in childhood (Hillman et al., 2011), as well as academic achievement (Alvarez-Bueno et al., 2017; Singh et al., 2018), and it may also reduce cognitive and motor decline in older adults (Bherer et al., 2013; Paillard, 2015; Roig et al., 2016).

Furthermore, a single bout of acute physical exercise can facilitate various cognitive functions, from executive functions like inhibition, verbal fluency, decision making, and stroop interference (Chang et al., 2012) to memory processes involving the encoding and consolidation of new information (Roig et al., 2013). Several reviews and meta-analyses (Etnier et al., 1997; Tomporowski, 2003; Chang et al., 2012; Roig et al., 2016; Loprinzi et al., 2019) have concluded that the overall effects are small, and are moderated by the duration, the intensity, the type, and the timing of the exercise.

Acute exercise increases one’s heart rate, which can contribute to achieve an optimal level of neurological and physiological arousal, supporting the engagement in cognitively demanding tasks (McMorris and Graydon, 2000; Audiffren et al., 2008). Furthermore, there seems to be a link between physical exercise and the release of several neurobiological substrates that may enhance memory processes, like neurotrophins or certain neurotransmitters (Chmura et al., 1994; Davranche et al., 2006; Roig et al., 2013). In this context, Winter et al. (2007) assessed peripheral levels of catecholamines (dopamine, epinephrine, and norepinephrine) and brain-derived neurotrophic factor (BDNF) of young adults before and after high intensity running, low intensity running, or a period of rest. Directly after the intervention, after 1 week, and after 8 months, participants took part in an associative vocabulary learning task. After high intensity running, participants were 20% faster in learning the vocabulary and showed the strongest increases in BDNF and catecholamine levels. Higher levels of BDNF were related to better short-term learning, whereas higher levels of dopamine correlated with intermediate- and epinephrine with long-term retentions of the new vocabulary.

Overall, acute physical exercise affects the release of different neurobiological substrates, which may moderate memory encoding (Winter et al., 2007) and memory consolidation (Cahill and Alkire, 2003; Chowdhury et al., 2012). Due to the time-dependent nature of these two memory processes, memory encoding can be mainly effected by physical exercise before or during encoding, while memory consolidation would be more strongly affected by a bout of physical exercise after encoding (Roig et al., 2016).

The majority of studies investigating the acute effects of exercise on memory processes asked participants to exercise either before or after memory encoding (Schramke and Bauer, 1997; Coles and Tomporowski, 2008; Pesce et al., 2009; Labban and Etnier, 2011; Etnier et al., 2014; Hötting et al., 2016; for a recent review, see Loprinzi et al., 2019). However, there is also an increasing body of research showing that acute exercise *during* memory encoding can enhance episodic memory performance (Schmidt-Kassow et al., 2010, 2013, 2014; Mavilidi et al., 2016; Liu et al., 2017).

Mavilidi et al. (2017) used physical exercise to teach 90 preschool children the names and positions of planets of our solar system. Children were either sitting, running laps around the room (task-unrelated physical activity), or they were running successively from the sun to the planets lying on the floor (task-related physical activity) while the teacher repeated the names of the planets. Although children in the task-related physical activity group had the highest memory scores in an immediate and a delayed retention test, the task-unrelated group, which performed a typical acute exercise while encoding, also had significantly higher memory scores, compared with the control group.

The positive effect of physical exercise during memory encoding could also be shown for language learning tasks. Liu et al. (2017) tested 40 Chinese-English L2-learners in a vocabulary learning task. The learning phase included eight test sessions with one session per week. Each to-be-learned list was presented to the participants three times per session. The list consisted of 40 picture-name pairs. During learning, participants were either seated or were bicycling at 60% of their maximum heart rate. After each session and 1 month after the last session, participants took part in a word–picture verification task and in a semantic judgment task. Already after the first training session, the physical exercise group remembered more correct picture-name pairs in the picture verification task, while it took several weeks before they outperformed the control group in the semantic judgment task. This result indicates that already a single bout of physical exercise can have a positive effect on vocabulary learning.

In a similar way, Schmidt-Kassow et al. (2010) tested 12 adult native German speakers in three sessions per week for 3 weeks. During each session, the participants listened twice to the same 80 French-German word-pairs while being seated (control group) or while cycling (physical exercise group) at moderate speed. After every third learning session, participants’ knowledge of the to-be-learned words was tested. The physical exercise group remembered more words compared to the control group. In a later study, Schmidt-Kassow et al. (2013) could replicate their results. They tested 105 German native speakers in a similar study design, except that this time there was an additional group that bicycled before memory encoding. Furthermore, participants learned a list of 80 Polish-German word-pairs, in only two learning sessions, and participants performed only two vocabulary learning tests. The results of the vocabulary learning tests revealed better memory performance for the group that cycled during encoding compared to the control group.

While the studies by Mavilidi et al. (2017) and Schmidt-Kassow et al. (2010, 2013, 2014) indicate that memory encoding in young children and in young adults can profit from concurrent exercise, findings for older adults appear less promising. To our knowledge, the effects of acute exercise *during* memory encoding in older adults have mainly been investigated in the context of cognitive-motor dual-task research. The general assumption in these study paradigms is that older adults have to invest more cognitive resources into seemingly automatized motor tasks like walking, and therefore show more pronounced performance decrements (dual-task costs) when a motor and a cognitive task have to be performed concurrently (for reviews, see Woollacott and Shumway-Cook, 2002; Schaefer, 2014). Studies by Lindenberger et al. (2000) and by Li et al. (2001) asked young and older adults to encode word lists (using a memory strategy) while walking on narrow tracks of different complexities. In these studies, older adults indeed showed pronounced performance reductions in memory when encoding took place while walking as opposed to sitting. However, the tracks used in these studies had been constructed to be rather challenging to walk on (narrow, sometimes with numerous turns, and sometimes including obstacles). It therefore remains unclear whether walking/jogging without such challenges has beneficial effects on memory in older adults.

Findings on acute exercise effects before or after a memory task in older adults are equivocal. Stones and Dawe (1993) asked elderly nursing home residents to perform a word fluency task (memory retrieval) before or after performing a 15-min non-strenuous exercise (intervention group), or after watching a video. Participants in the exercise group retrieved more category exemplars following exercise than the control group. In addition, Segal et al. (2012) reported post-learning exercise to enhance memory consolidation in older adults with and without mild cognitive impairment. However, Schramke and Bauer (1997) did not find improvements in the learning of word lists following exercise in young and older adults compared to a resting condition.

The current study investigates lifespan differences in the acute effects of exercise on vocabulary learning. We tested children, young adults, and older adults in a vocabulary learning task. Subjects were recruited via a local basketball club, and were all experienced basketball players. The participants learned pairs of words and pseudo-words, either while standing, while running, or while running and dribbling a basketball. Based on previous reports of enhanced vocabulary learning while exercising (Schmidt-Kassow et al., 2010, 2013, 2014), the positive influence of acute physical exercise on establishing an optimal physiological arousal (McMorris and Graydon, 2000; Audiffren et al., 2008), and the link between exercise and the release of neurobiological substrates that enhance memory processes (Chmura et al., 1994; Davranche et al., 2006; Roig et al., 2013), we hypothesized that more words are remembered when encoding takes place while running as compared to standing in all age groups. Furthermore, we assume that running while dribbling a basketball (as compared to running only) exerts an additional cognitive load. The cognitive load theory (see Sweller et al., 1998, 2019) distinguishes three types of cognitive load. *Intrinsic cognitive load* refers to the complexity of the information being processed, while considering the knowledge of the person processing the information. *Extraneous cognitive load* includes the instructional procedures that may reduce or increase cognitive load. *Germane cognitive load* describes the resources available to deal with intrinsic cognitive load (Sweller et al., 2019). In this context, dribbling a basketball is expected to increase the extraneous cognitive load, since it is a complex motor skill requiring attentional resources. However, motor expertise is influenced by experience and age: Younger adults should be able to compensate for the additional task with their experience in basketball dribbling, having reached a stage of complete automation of the motor task (Fitts and Posner, 1967). Children, on the other hand, still have to devote some attentional resources into the dribbling task, and may have fewer resources overall. And older adults have to compensate for aging-related declines in sensory and motor systems, as assumed by the dual-task literature (Kahneman, 1973; Navon and Gopher, 1979; Schaefer, 2014). We therefore predict that running while dribbling a basketball reduces the number of words remembered in children and older adults, but not in young adults. Lastly, we assume that all age groups will improve from recall 1 to recall 3, which reflects the learning process.

## Methods

### Participants

A statistical *a priori* power analysis was performed for sample size estimation (GPower 3.1.9.2). According to the current literature (Schmidt-Kassow et al., 2013, 2014), we assumed a large effect size (*f* = 0.40) with an α = 0.05 and power = 0.80 for the estimated main effect of condition, which resulted in a suggested sample size of *N* = 66. Twenty-four children (*M*_*age*_ = 12.3 years; 13 girls), 30 young adults (*M*_*age*_ = 21.5 years; 17 women), and 24 older adults (*M*_*age*_ = 59.3 years; 9 women) were recruited and tested in a recreational Basketball club at the Saarland. All of them had normal or corrected-to-normal vision and hearing. The declaration of consent was signed by all participants, in case of children by their legal guardian. The study was approved by the Ethics committee of Saarland University. The current study has been preregistered under the following link: https://aspredicted.org/blind.php?x=3qf4yt.

### Cognitive Background Variables

To allow for comparisons across the three age groups, tests for cognitive speed (Digit Symbol Substitution task, Wechsler, 1981), sustained attention (D2 Test, Brickenkamp et al., 2010), and knowledge of German vocabulary (MWT-A questionnaire, Lehrl et al., 1991) were measured and used as background variables. Consistent with the developmental literature (Li et al., 2004), young adults outperformed children in cognitive speed, while older adults showed the highest scores in the test for knowledge of German words (see Table 1 for descriptives and cognitive background information).

**TABLE 1:** Descriptives, cognitive background information, and reliability of the vocabulary learning task.

Group	Children (C)	Young adults (YA)	Older adults (OA)	ANOVA*
*n* Age (years)	24 (*f* = 13) *M* = 12.3 SD = 1.3	30 (*f* = 17) *M* = 21.5 SD = 4.4	24 (*f* = 9) *M* = 59.3 SD = 5.1	

Basketball experience (years) Training per week (number of sessions) Still playing actively (percentage)	*M* = 4.2 SD = 1.9 *M* = 3.3 SD = 1.3 100%	*M* = 11.8 SD = 5.3 *M* = 3.5 SD = 1.2 100%	*M* = 40.5 SD = 5.4 *M* = 1.1 SD = 0.2 46%	OA > YA > C, *F*(2,50) = 244.20, *p* < 0.001 YA = C > OA, *F*(2,54) = 18.71, *p* < 0.001

Digit symbol substitution (cognitive speed; items per second)	*M* = 0.5 SD = 0.1	*M* = 0.7 SD = 0.2	*M* = 0.5 SD = 0.1	YA > C = OA, *F*(2,75) = 16.71, *p* < 0.001

D2 (sustained attention, score)	*M* = 114.0 SD = 29.7	*M* = 120.4 SD = 19.4	*M* = 136.3 SD = 42.8	OA = YA > C, *F*(2,75) = 3.25, *p* < 0.05

MWT-A (vocabulary, number of correct solutions)	*M* = 21.4 SD = 4.0	*M* = 28.2 SD = 3.2	*M* = 31.9 SD = 1.7	OA > YA > C, *F*(2,75) = 68.15, *p* < 0.001

Immediate recall accuracy [%] German word (GW) Pseudo word (PW)	93.19 87.71	97.61 92.00	94.72 85.28	GW > PW, *F*(1,75) = 56.54, *p* < 0.001 No sign. main effect of age group, *F*(2,75) = 2.72, *p* = 0.073, or age group × language interaction, *F*(2,75) = 1.97, *p* = 0.147

Retest reliability for recall time 1 to recall 3 (Cronbach’s Alpha)	α = 0.882	α = 0.946	α = 0.885	

*^∗^Significant effects have been further analyzed with independent-samples *t*-tests.*

### Experimental Task

#### Vocabulary Learning Task

The Vocabulary Learning task was constructed based on extensive piloting of the paradigm. Measures of retest reliability are depicted in Table 1. The goal of the task was to learn a list of 20 new pseudo-words. Each pseudo-word corresponded to a German word. Pseudo-words were constructed using syllables that can be pronounced in the German language (examples of pseudo words: kebruli, curlef, and ogizav). A word-pair was presented for 6 s on a large screen (for example, Wolf – kebruli) to make sure that participants of each age group had enough time to read the word-pair. Then participants had 7 s to encode the word-pair, without seeing it any more on the screen. We argue that this time interval has to be used for active encoding processes (i.e., continuous rehearsal), since participants had to enter both words using a computer keyboard immediately afterward. Immediate recall accuracies were very high in all age groups (see Table 1). After 15 s, which was enough time to enter the word-pair and to refocus on the screen, the next word-pair was presented, until all word-pairs of the list had been presented. A sound signal indicated when the 6 s presentation time, the 7 s encoding time, and the 15 s enter time ended.

**FIGURE 1:**
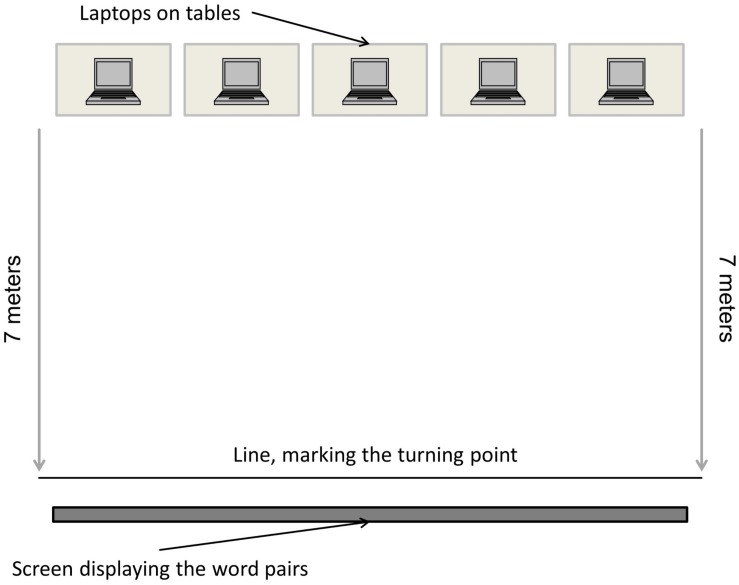
Experimental setup of the vocabulary learning task.

In a between-subjects design, the 7 s of encoding took place either while standing, while running, or while running and dribbling a basketball. The distance in both running conditions was 7 m forth and 7 m back to where the Laptops stood (see Figure 1 for the experimental setup). The distance of 14 m was chosen to induce a moderate running speed, which was adapted to the encoding time of 7 s.

Following the 20 word pairs of each list, participants took part in a cued recall test, where only the German words were presented and the participants had to enter the corresponding pseudo-words. During recall, German cue words were presented one at a time in randomized order. Participants had no time limit to answer the cued recall test. Two additional encoding and recall trials were administered in the testing session, using the same word-pseudo-word pairs, but always in randomized order, resulting in three repetitions of the to-be-learned lists.

On the following day, each participant received an email with a link to a fourth cued recall test via internet. The dependent variable for memory was the number of correctly spelled pseudo-words in each cued recall test. Spelling errors were not scored as a remembered word.

### Procedure

Before data collection started, each participant’s identification number was randomly assigned to one of the three treatment conditions (standing, running, and dribbling) using a random algorithm of the program Microsoft Excel. Participants were tested in groups with up to five participants completing the same condition. The test session lasted for about 1 h and started with the completion of the demographic questionnaire, that included questions about age, sex, and sport activities. Then each participant was assigned to one of the five laptops standing on the tables. The test administrator explained the task and how the words had to be entered in the laptop. Then participants took part in a practice phase with one word-pair until everyone got used to the timing of the encoding phase. For participants in the running and dribbling conditions, the practice phase was also used to accustom participants to the required running speed. We did not observe any dribbling mistakes throughout the entire study. After practice, the first 20 word-pairs were presented. Following every cued recall test, participants completed one of the three background tasks (after trial one: Digit Symbol Substitution; after trial two: MWT-A; after trial three: D2). Working on the cognitive tasks between the trials led to a schedule of distributed rather than massed practice for the memory-encoding task within a testing session. On the following day, participants were reminded via text messages to fill in the cued recall test one more time (internet based assessment).

### Data Analysis

The vocabulary learning task was analyzed with a mixed-design analyses of variance (ANOVA) with position (recall 1–4) as within-subjects factor and age group (3: children, young adults, and older adults) and condition (3: standing, running, and running and dribbling) as between-subjects factors. *F* values and partial Eta square values for effect sizes are reported. If sphericity assumptions were violated, Greenhouse–Geisser corrected values are reported. The alpha level used to interpret statistical significance was *p* < 0.05. Significant main effects were further investigated by planned *t*-tests with Bonferroni corrected levels of significance. If the *a priori* Levene-Test was violated, values for *t*-tests with unequal variances are reported. For paired-samples *t*-tests, we present Cohen’s *d*_*z*_ effect sizes and for independent samples *t*-tests, we present Cohen’s *d* effect sizes (Cohen, 1988).

## Results

### Vocabulary Learning Task

**FIGURE 2:**
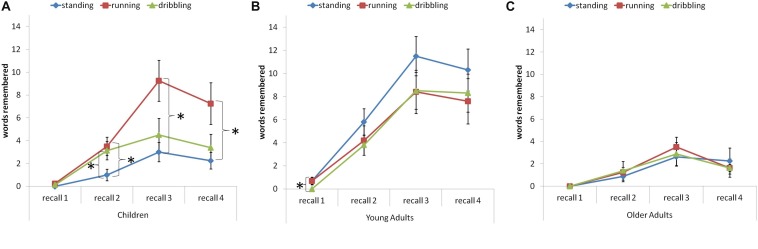
Memory performance in the vocabulary learning task of children **(A)**, young adults **(B)**, and older adults **(C)** at recall test 1 to 4, in the standing, running, and dribbling conditions. Asterisks indicate results with *p* < 0.05. Error bars = SE means.

The ANOVA with position (4) as within-subjects factor and age group (3: children, young adults, and older adults) and condition (3: standing, running, and running and dribbling) as between-subjects factors was conducted to investigate the effects of acute physical exercise on vocabulary learning. Figure 2 depicts the pattern of findings.

The results of the ANOVA show a significant main effect of position, *F*(1.58,109.19) = 109.820, *p* < 0.001, η*^2^p* = 0.614. Paired-samples *t*-tests (Bonferroni corrected level of significance to *p* < 0.008) indicate significant performance increases during the learning session from recall 1 to recall 3, followed by a performance reduction comparing recall 3 with recall 4 (see Table 2). There was a significant main effect of age group, *F*(2,69) = 16.588, *p* < 0.001, η*^2^p* = 0.325. Independent samples *t*-tests indicate that children (*M* = 3.14, SD = 2.67) remembered more words than older adults (*M* = 1.52, SD = 1.51), *t*(46) = 2.583, *p* < 0.02, *d* = 0.75, but fewer words than younger adults (*M* = 5.82, SD = 3.67), *t*(52) = 3.105, *p* < 0.01, *d* = 0.82. Young adults remembered more words than older adults, *t*(52) = 5.825, *p* < 0.001, *d* = 1.47. The results showed no main effect of condition, *F*(2,69) = 0.59, *p* = 0.560, η*^2^p* = 0.017. Furthermore, the results show a significant interaction of position and age group, *F*(3.17,109.19) = 13.771, *p* < 0.001, η*^2^p* = 0.285. This interaction can be explained by paired-samples *t*-tests (Bonferroni corrected level of significance to *p* < 0.008) showing that children’s and older adult’s memory performance decreases from recall 3 to recall 4, but young adult’s performance does not. In addition, children’s and older adult’s performance does not significantly differ between recall 2 and recall 4, whereas young adult’s performance does improve. The results show no interaction of position and condition, *F*(3.17,109.19) = 0.885, *p* = 0.456, η*^2^p* = 0.025, and no interaction of condition and age group, *F*(4,69) = 2.173, *p* = 0.081, η*^2^p* = 0.112, but we see a significant three-way interaction of position, age group, and condition, *F*(6.33,109.19) = 2.302, *p* < 0.05, η*^2^p* = 0.118. Paired-samples *t*-tests show that children remembered more words in the running condition compared to the standing condition at recall 2, *t*(14) = 2.646, *p* < 0.02, *d* = 1.32, recall 3, *t*(9.97) = 3.157, *p* < 0.02, *d* = 1.58, and recall 4, *t*(9.15) = 2.54, *p* < 0.04, *d* = 1.27. Comparing the dribbling condition and the standing condition, they only remembered more words at recall 2, *t*(14) = 2.229, *p* < 0.05, *d* = 1.12 (see Figure 2A). For the young adults, there was only one difference between the running and dribbling condition at recall test 1, *t*(9) = 2.33, *p* < 0.05, *d* = 1.04 (see Figure 2B), and there were no differences between conditions for the older adults at any recall test (see Figure 2C).

**TABLE 2:** Follow-up analysis of the significant main effect of position with paired-samples *t*-tests.

Comparisons	Difference of the means	*SD*	*t*	*p*
Recall 1 with rec. 2	–2.67	2.63	–8.949	<0.001
Recall 1 with rec. 3	–6.04	4.89	–10.913	<0.001
Recall 1 with rec. 4	–5.00	4.90	–9.004	<0.001
Recall 2 with rec. 3	–3.37	3.05	–9.778	<0.001
Recall 2 with rec. 4	–2.33	3.09	–6.678	<0.001
Recall 3 with rec. 4	1.04	1.83	–5.021	<0.001

## Discussion

The current study aimed to investigate if physical exercise during vocabulary learning can enhance memory performance of children, young adults, and older adults. We hypothesized that memory performance would profit from the running condition in all age groups. This hypothesis could be partially confirmed. Only children’s memory performance benefited from running during memory encoding, whereas young and older adults’ performances were comparable for standing and running conditions.

But why did only children profit from physical exercise, and not young or older adults? In the current study, all age groups ran at the same speed in order to keep the running distance and the encoding time comparable between the age groups. It is possible that younger adults did not benefit to the same extent as children since children may have experienced a higher physical exertion at the same running speed. Exercise-induced changes in arousal and neurobiological substrates may have been too low in the young adult group, and a higher intensity (faster running speed) could have changed the pattern of results (Chang et al., 2012). However, recent studies have shown that even walking at a preferred speed can facilitate memory performance (Schmidt-Kassow et al., 2014).

Another explanation could be that running has interfered with the use of cognitive strategies, like rehearsal (actively repeating information), deep encoding (linking the information with associations or images), or clustering (organizing new information into related groups). In reference to the cognitive load theory (Sweller et al., 2019), younger adults should have a larger knowledge base to connect the to-be-learned words with in their long-term memory. They can then use their working memory to embed the new words into pre-existing semantic structures via deep encoding, and such processes may work best when not being distracted by physical activities. In fact, it seems as if young adults were most successful in memory encoding while standing, even if this trend does not reach significance in the current study. Compared to adults, children do not consistently use cognitive strategies yet (Ornstein, 1978; Schneider, 2008). Instead, the structure and rhythm imposed by the running condition may have helped them to engage in memory strategies like continuous rehearsal. In future research, the use of cognitive strategies should be taken into account, for example by instructing participants to use a specific strategy like the method-of-loci, which integrates the new information into visualizations of familiar places (Li et al., 2001). A thorough monitoring of exercise intensity and physical exertion using heartrates or physical exertion questionnaires would help to interpret future findings.

For older adults, we did not find any differences between the three conditions. Overall, the memory performance of this age group was very poor. On average they only remembered three words (SD = 2.50) out of 20 possible words at their best recall test (recall test 3). These results indicate that the task-difficulty of the memory task was too high for older adults, making it difficult to interpret the current results. Dual task studies using walking and memory encoding in older adults would even have predicted memory performance decrements (Lindenberger et al., 2000; Krampe et al., 2011; Schaefer et al., 2014), but these studies used rather challenging walking conditions like virtual worlds, narrow tracks, or obstacles. Future studies on memory encoding while exercising should try to avoid floor or ceiling effects in the motor or memory task. It could be worthwhile to adjust task-difficulties individually, or at least on the level of age groups.

Based on the motor learning and expertise literature (Fitts and Posner, 1967; Schaefer, 2014), we expected children’s and older adults’ memory performance to decrease in the dribbling condition, compared to the running condition, while young adults should be able to keep their memory performance stable. Our expectations could partially be confirmed. As expected, the results show no decrease in memory performance in the young adult group. However, contrary to our expectations, older adults also did not show memory performance decrements while dribbling. Again, this may be due to the excessive demands that older adults have been confronted with. Children showed comparable performance levels for standing and dribbling at the later stages of the study. Since all of our participants were experienced basketball players, running and dribbling a basketball may have been automatized even in our youngest participants already. Not all of the elderly participants were still playing basketball regularly. This lack of current practice of a motor skill could lead to de-automatization processes, making it more difficult to profit from certain types of exercise while learning. Future research should investigate motor expertise more systematically. Since a lack of power might have been responsible for not finding clearer effects in our study, future research should also test larger sample sizes and consider using within-subjects designs.

Another issue that is worth investigating is the exact timing of exercise and memory encoding. Beneficial effects of exercise on memory could be even larger if the exercise bout takes place before or after memory encoding (Chang et al., 2012; Roig et al., 2013; Loprinzi et al., 2019). Roig et al. (2016) argued that memory encoding may be more strongly affected by exercise before encoding, while memory consolidation may more affected by exercise after memory encoding. On the other hand, it may be possible that physical exercise during encoding is particularly appropriate for enhancing both memory processes, by optimizing arousal level throughout the entire learning phase.

The current study provides an example of how physical exercise could be implemented in other learning activities (e.g., vocabulary learning) (see Diamond and Ling, 2016, 2018; Hillman et al., 2018 for an ongoing discussion on physical activity and its effects on executive functions). The results of the current study show that the integration of physical exercise into memory encoding can be particularly beneficial for children. Using physical exercise that is meaningfully related to the cognitive task may lead to stronger effects (see Mavilidi et al., 2016, 2017). We conclude that the combination of physical and mental activities has power to improve learning while at the same time giving students the opportunity to be physically active. Schools should provide optimal learning environments and support students in acquiring efficient learning strategies. Physical exercise may contribute to achieve this goal by enhancing cognitive activation (Pesce et al., 2009) and thus facilitating the learning process stimulated by the school.

## Data Availability Statement

The datasets generated for this study are available on request to the corresponding author.

## Ethics Statement

The studies involving human participants were reviewed and approved by the Ethics Committee of Saarland University. Written informed consent to participate in this study was provided by the participants’ legal guardian/next of kin.

## Author Contributions

Both authors contributed to the study design and cooperated in conducting the literature review. GA analyzed and interpreted the data, with input from SS and led the drafting of the manuscript, with substantial contributions from SS.

## Conflict of Interest

The authors declare that the research was conducted in the absence of any commercial or financial relationships that could be construed as a potential conflict of interest.
